# Supplementary material for: Thermostable ancestors enabled evolutionary diversification of promiscuous chemical defence enzymes
Source: EMBO J. 2026 Mar 26;45(9):2938–64. doi: 10.1038/s44318-026-00699-y (PMC13144609; doi:10.1038/s44318-026-00699-y)
Supplement: Supplementary file 1 — Appendix [file 44318_2026_699_MOESM1_ESM.pdf]

Appendix for:

Thermostability in vertebrate ancestors of promiscuous chemical defence enzymes facilitated evolutionary diversification

Raine E. S. Thomson[1], Yosephine Gumulya[1], Anthony W. Bengochea[1], Gabriel Foley[1], Julian Zaugg[2], Anna Aagaard[3], Connie M. Ross[1], James Beckett[1], Mikael Bodén[1], Ulrik Jurva[3], Martin A. Hayes[4], Shalini Andersson[4], Elizabeth M.J. Gillam[1]*

[1] School of Chemistry and Molecular Biosciences, The University of Queensland, St. Lucia, Brisbane, 4072 Australia

[2]Australian Centre for Ecogenomics, School of Chemistry and Molecular Biosciences, The University of Queensland, St Lucia, QLD 4072, Australia

[3] Drug Metabolism and Pharmacokinetics (DMPK), Research and Early Development, Cardiovascular, Renal and Metabolism (CVRM), BioPharmaceuticals R&D, AstraZeneca, Gothenburg, Sweden

[4] Discovery Sciences, BioPharmaceuticals R&D, AstraZeneca, Gothenburg, Sweden.

*Corresponding author: e.gillam@uq.edu.au

Table of Contents: **p.**

1. Appendix Table S1. Percentage amino acid identity between all extant and ancestral forms assessed. 4-5
2. Appendix Table S2. Comparison of joint and marginal inferences: average posterior probabilities and percent identities of alternative joint and marginal inferences at each ancestral node characterized. 6-8
3. Appendix Table S3. Expression yield (nmol/L culture) and thermostability ($^{60}T_{50}$ and $^{10}T_{50}$) of recombinant ancestral and extant P450s. 9-10
4. Appendix Table S4. Analysis of the correlation between the Kyte-Doolittle hydrophobicity index and thermostability performed using a phylogenetic independent contrast method 10
5. Appendix Table S5. Percentage activity observed with the addition of ligands. 11
6. Appendix Table S6. Binding analysis of the interaction of ancestors with five representative ligands assessed by perturbation of the haemoprotein spectrum. 12-13
7. Appendix Table S7. Effect of tree topology *vs.* evolutionary rate on the sequence identity of inferred ancestors. 14-15
8. Appendix Table S8. Comparison between the sequences of the resurrected ancestral proteins and the respective consensus sequences for equivalent clades 16
9. Appendix Figure S1. Analysis of CYP2CEH thermostability by circular dichroism. 17

10. Appendix Figure S2. Kyte-Doolittle hydrophobicity of buried and exposed residues in specific structural elements. 18-19

11. Appendix Figure S3. Metabolism of testosterone by the CYP2 extant and ancestral enzymes. 20-21

12. Appendix Figure S4. Conversion of coumarin to umbelliferone (7-hydroxycoumarin) by the CYP2 extant and ancestral enzymes. 22

13. Appendix Figure S5. *O*-Dealkylation of fluorogenic 7-alkoxyresorufin derivatives by CYP2 extant and ancestral enzymes. 23-24

14. Appendix Figure S6. Metabolism of P450-Glo^{TM} luciferin derivatives by ancestral and extant CYP2 enzymes. 25-26

15. Appendix Figure S7. Indigo formation by ancestral CYP2s. 27

16. Appendix Figure S8. Effect of alternative ligands on the activity of ancestral CYP2 forms. 28

17. References for Appendix 29

Appendix Table S1. Percentage amino acid identity between all extant and ancestral forms assessed.

Identity was calculated based on sequences used for expression, including N-terminal modifications.

	2a5	2A6	2A13	2B6	2C8	2C9	2C11	2C18	2C19	2E1	2F3	2S1	2A	2B	2C	2E	2F	2S
2a5	100																	
2A6	85	100																
2A13	89	94	100															
2B6	52	52	54	100														
2C8	50	48	50	50	100													
2C9	50	48	49	48	77	100												
2C11	50	50	52	49	71	73	100											
2C18	50	48	49	48	78	81	74	100										
2C19	50	49	51	46	75	88	73	78	100									
2E1	46	45	47	45	55	55	57	55	58	100								
2F3	51	51	51	50	51	52	50	52	52	49	100							
2S1	46	47	47	47	41	42	42	41	42	38	45	100						
2A	91	89	92	55	50	50	53	50	54	50	52	48	100					
2B	62	61	62	75	56	55	59	54	58	53	56	50	65	100				
2C	58	57	58	51	70	74	73	73	77	65	54	44	61	64	100			
2E	54	55	54	51	60	61	63	61	63	79	50	42	58	61	76	100		
2F	57	57	57	51	52	52	54	54	56	51	81	46	60	63	61	55	100	

2S	57	57	57	58	48	50	51	48	53	46	54	69	61	70	56	54	60	100	2BS					
2BS	66	65	66	67	57	58	60	57	61	54	59	57	69	86	68	63	67	81	100	2CE				
2CE	61	61	62	56	66	66	67	67	69	63	57	46	65	68	84	74	65	61	75	100	2ABGS			
2ABGS	68	67	68	62	61	60	63	60	63	57	60	55	72	78	73	65	69	73	91	81	100	2CEH		
2CEH	65	63	64	58	63	62	64	64	65	60	61	50	68	72	78	70	70	66	81	90	88	100	2ABGSFT	
2ABGSFT	65	64	65	60	62	61	64	62	64	58	63	52	69	74	75	68	73	68	84	85	92	95	100	2ABGSFTCEH
2ABGSFTCEH	64	63	64	59	62	61	64	63	64	59	62	51	68	73	76	69	72	67	83	87	91	96	98	100

Appendix Table S2. Comparison of joint and marginal inferences: average posterior probabilities and percent identities of alternative joint and marginal inferences at each ancestral node characterized.

	Average Percent Probability [a] ± SD		Joint vs Marginal [b]		
	Joint	Marginal	Percent Identity	Differences	Substitutions (joint/marginal)
N4_2ABGSFTCEH	96.3+-3.2	96.9+-3.2	97.98	10 (8)	(T/S; R/K) H/Q; **Y/C**; E/D; H/Q; A/T; E/D; I/L; L/V
N5_2CEH	95.3+-3.7	95.8+-3.7	98.18	9 (7)	(T/S; S/K) I/L; H/Q; I/F; V/I; **Y/C**; L/M; H/Q
N8_2CE	95.5+-3.6	96.1+-3.6	97.57	12 (11)	(R/K) Q/E; H/Q; I/F; V/I; **Y/C**; N/S; K/Q; H/Q; I/V; S/T; **E/I**
N10_2C	96.0+-3.5	96.2+-3.5	98.79	6 (5)	(G/S) **A/K**; R/K; F/I; M/I; V/I
N357_2E	94.8+-4.1	94.9+-4.1	98.98	5 (4)	(I/V) V/I; L/I; H/Q; L/V
N559_2ABGSFT	96.4+-3.2	96.9+-3.2	98.18	9 (7)	(T/S; R/K) L/M; E/D; V/I; H/Q; A/S; E/D; I/L

N562_2F	95.6+-3.8	95.9+-3.8	98.78	6 (4)	(L/V; **T/R**) E/Q; V/I; E/D; I/V
N623_2ABGS	95.9+-3.2	96.9+-3.3	97.77	11 (11)	**R/T**; L/M; F/L; E/D; R/N; E/D; A/T; H/Q; A/S; S/A; L/F
N624_2BS	91.9+-4.8	92.6+-4.8	96.36	18 (16)	(A/C; K/R) A/T; **R/T**; Q/R; **E/A**; R/N; **R/G**; S/N; A/S; S/A; A/S; A/T; K/R; H/R; L/I; S/A; **T/P**
N625_2B	94.2+-4.2	94.4+-4.2	98.78	6 (5)	(Q/H) K/R; **S/P**; N/D; T/N; **T/I**
N727_2S	91.3+-5.1	91.6+-5.1	98.18	9 (6)	(K/R; **T/R; R/S**) Q/H; R/H; K/Q; S/E; A/T; K/Q;
N851_2A	98.9+-2.0	98.9+-2.0	99.60	2 (2)	N/K; N/S

[a] The average percent probability was calculated by summing the probability of each amino acid of a given ancestor at each position and dividing by the number of positions. The average probability can be interpreted a number of different ways (Sennett and Theobald 2023): it represents the fraction of positions which are expected to have been inferred correctly corresponding to a hypothetical true ancestor. It also represents the expected identity any randomly sampled sequence from the distribution would have to the relevant sequence. The standard deviation therefore represents the range of likely identity values to that ancestor. For example, for the joint N4_2ABGSFTCEH ancestor, a random sample of the marginal distribution would on average have an identity of 96.3 with joint N4, and most sequences would be within ~93.1

and ~99.5 % identical to joint N4. This data shows that the joint and marginal reconstructions are very similar, and generally closer to each other than a random sample of the marginal distribution would be expected to be.

[b] The joint and marginal ancestors inferred at each position in the phylogenetic tree were compared in terms of net percent identity and number of amino acid differences across the whole protein sequence. Numbers in parentheses indicate the number of differences excluding any within the N-terminal membrane anchor sequence which was removed for expression, so would not count towards difference in catalytic activity between the alternative expressed ancestors.

[c] The residues that differ between joint and marginal ancestors are shown. Those in parentheses and grey text are within the N-terminal membrane anchor sequence which was removed for expression, so would not count towards difference in catalytic activity between the alternative expressed ancestors. Positions which differed generally aligned between ancestors at different positions of the phylogenetic tree. Non-conservative substitutions are shown in bold.

Appendix Table S3. Expression yield (nmol/L culture) and thermostability ($^{60}T_{50}$ and $^{10}T_{50}$) of recombinant ancestral and extant P450s.

CYP Variant	Expression (nmol/L) [a]	$^{10}T_{50}$ (°C) [a, b]	$^{60}T_{50}$ (°C) [a, b]
2a5	540 ± 40	43 ± 1[d]**	43 ± 2*
2A6	650 ± 90	46 ± 2*	43.3 ± 0.4**
2A13	500 ± 100	43.6 ± 0.3****	41 ± 1**
**2A_N851**	**2000 ± 100**	**54.1 ± 0.5******	**49 ± 1*****
2B6	70 ± 10	48 ± 1*	45 ± 2**
**2B_N625**	**170 ± 10**	**55.1 ± 0.2******	**52 ± 1*****
2C8	400 ± 100	46.9 ± 0.2***	43 ± 0.6**
2C9	240 ± 80	43.6 ± 0.9***	42 ± 1**
2C11	260 ± 40	44 ± 2****	41 ± 1**
2C18	270 ± 40	56 ± 1***	51.1 ± 0.6*
2C19	470 ± 30	55 ± 1***	50 ± 2**
**2C_N10**	**240 ± 20**	**75 ± 2***	**68 ± 3***
2E1	1100 ± 100	48.2 ± 0.5***	44.9 ± 0.3*
**2E_N357**	**800 ± 200**	**53 ± 0.5*****	**51 ± 2****
2F3	590 ± 20	48.2 ± 0.6**	44 ± 1*
**2F_N562**	**560 ± 30**	**53 ± 1******	**49 ± 2****
2S1	700 ± 20	53 ± 1**	48.6 ± 0.5***
**2S_N727**	**860 ± 200**	**61.3 ± 0.6****	**53.5 ± 0.3***
**2BS_N624**	**460 ± 30**	**64.8 ± 0.5*****	**59 ± 1****
**2CE_N8**	**820 ± 90**	**80 ± 1***	**76.8 ± 0.6*****
**2ABGS_N623**	**560 ± 20**	**74.4 ± 0.3**	**68.9 ± 0.6**

**2CEH_N5**	**220 ± 40**	**85.9 ± 0.2****	**82.9 ± 0.8***
**2ABGSFT_N559**	**160 ± 30**	**72.9 ± 0.9**	**68.5 ± 0.3**
**2ABGSFTCEH_N4**	**250 ± 60**	**75 ± 1**	**71 ± 3**

[a]Data represent the mean ± SD of three (thermostability, extant expression) or four (ancestor expression) biological replicates.

[b]T_{50} values were interpolated by fitting a sigmoidal curve to the stability profiles shown in Figure S1.

[c]Ancestral forms are shown in bold.

[d]Asterisks indicate significantly different T50 to the immediate ancestor: *, $p<0.05$; **, $p<0.01$, ***, $p<0.001$; ****, $p<0.0001$, two tailed, heteroscedastic Student's *t*-test.

Appendix Table S4. Analysis of the correlation between the Kyte-Doolittle hydrophobicity index and thermostability performed using a phylogenetic independent contrast method

Comparison	Correlation	p-value
Buried vs $^{10}T_{50}$ (°C)	0.509	0.110
Buried vs $^{60}T_{50}$ (°C)	0.477	0.138
Exposed vs $^{10}T_{50}$ (°C)	0.072	0.834
Exposed vs $^{60}T_{50}$ (°C)	0.243	0.471

Appendix Table S5. Percentage activity observed with the addition of ligands.

The heatmap shows the relative percentage (%) catalytic activity of ancestral CYP2s towards probe substrates in the presence of 50 µM of the indicated compound, where N=1. The colour scale reflects the quantitative change in activity from the greatest relative inhibition (green) to greatest relative activation (red).

	2A_N851	2B_N625	2C_N10	2E_N357	2F_N562	2S_N727	2BS_N624	2CE_N8	2ABGS_N623	2CEH_N5	2ABGSFT_N559	2ABGSFTCEH_N4
Acetaminophen	79	100	71	98	79	104	95	70	83	87	49	69
Aminopyrine	66	80	86	67	104	98	98	72	84	99	64	76
Amlodipine	42	21	9	41	89	26	0	24	23	22	12	15
Amodiaquine	73	71	65	63	83	103	50	66	56	106	64	87
Atorvastatin	91	20	56	60	89	81	47	70	83	74	59	69
Benzbromarone	68	10	5	41	13	16	0	24	117	29	8	9
Carbamazepine	79	39	138	111	84	80	44	127	67	93	73	83
Celecoxib	67	55	61	54	89	61	68	90	89	92	68	27
Chlorzoxazone	64	63	90	73	100	43	15	92	19	78	76	65
Clopidogrel	69	2	154	44	79	20	53	97	32	56	56	53
Clozapine	80	73	39	51	92	15	86	62	73	67	60	46
Dextromethorphan	80	48	498	48	98	94	88	122	87	98	92	86
Diclofenac	72	34	60	74	109	54	36	76	72	58	63	52
Diltiazem	66	39	69	49	85	66	74	66	62	85	81	71
Donepezil	84	73	57	68	68	68	95	52	57	75	66	73
Ethinylestradiol	82	30	4	61	74	52	34	12	36	10	40	35
Erythromycin	82	55	73	65	110	71	85	64	52	88	83	84
Fluoxetine	90	23	21	37	81	76	99	24	59	50	50	40
Flutamide	107	35	54	45	83	48	36	72	27	68	41	45
Furosemide	90	81	79	49	111	70	87	52	82	84	66	85
Indomethacin	113	89	52	67	80	60	34	49	46	75	44	81
Imipramine	99	39	57	21	104	90	40	90	56	59	31	81
Levofloxacin	84	75	82	105	119	76	106	92	89	93	49	88
Metoprolol	66	85	70	86	94	79	108	72	112	103	80	89
Midazolam	67	14	15	81	113	39	13	7	63	28	45	39
Nevirapine	90	45	65	87	116	74	101	33	74	100	56	76
Olanzapine	79	64	40	53	116	64	57	40	79	98	61	50
Omeprazole	87	44	113	52	108	32	94	58	112	93	67	61
Pioglitazone	89	49	86	94	105	88	53	53	100	115	118	96
Pravastatin	89	77	87	77	94	72	91	48	84	116	79	87
Procainamide	97	120	82	86	113	108	80	80	105	86	93	94
Propranolol	92	94	71	75	110	108	77	62	86	92	82	96
Ritonavir	92	62	58	52	70	90	26	38	101	83	42	59
Rosiglitazone	88	74	30	21	24	70	35	49	58	65	59	55
Sulfamethoxazole	87	107	77	49	84	91	69	120	77	103	91	98
Tacrine	78	86	93	82	94	109	47	48	82	91	68	75
Tamoxifen	102	70	154	102	93	97	79	71	66	98	96	86
Testosterone	66	14	166	114	110	68	30	72	76	80	77	78
Ticlopidine	80	2	337	75	79	32	32	84	48	47	68	83
Tienilic acid	72	54	66	97	95	29	30	65	53	97	60	80
Valproic acid	78	71	85	88	63	94	80	61	64	94	72	74
Valsartan	83	64	90	76	113	46	90	57	70	92	85	100
Verapamil	81	69	60	64	70	74	72	80	73	91	48	77
Warfarin	86	65	104	71	78	56	31	81	62	104	41	98
Warfarin R	85	43	108	79	88	62	28	72	56	117	54	92
Warfarin S	86	55	104	73	92	34	56	80	74	128	66	90
Zafirlukast	53	44	35	84	28	10	24	29	83	36	41	17
Zomepirac	79	41	94	66	90	82	24	74	67	84	74	75

Appendix Table S6. Binding analysis of the interaction of ancestors with five representative ligands assessed by perturbation of the haemoprotein spectrum.

Enzyme	Celecoxib		Clopidogrel		Ritonavir		Testosterone		Ticlopidine	
	K_d (µM)	ΔA_{max}	Kd(µM)	ΔA_{max}	Kd(µM)	ΔA_{max}	Kd(µM)	ΔA_{max}	Kd(µM)	ΔA_{max}
**2A**	weak [a]	weak	weak	weak	34 ± 2	0.0361 ± 0.0009	63 ± 16	0.014 ± 0.001	5 ± 3	0.007 ± 0.0009
**2B**	24 ± 13b[a]	0.014 ± 0.002	0.09 ± 0.08	0.026 ± 0.002	3.6 ± 0.6	0.043 ± 0.001	167 ± 23	0.042 ± 0.002	4 ± 1	0.029 ± 0.002
**2C**	4 ± 2	0.0107 ± 0.0008	3 ± 1	0.0077 ± 0.0005	13 ± 4	0.015 ± 0.001	21 ± 6	0.0060 ± 0.0003	31 ± 19	0.005 ± 0.001
**2F**	weak	weak	6 ± 4	0.009 ± 0.002	22 ± 8	0.053 ± 0.006	weak	weak	6 ± 2	0.018 ± 0.001
**2S**	weak	weak	3 ± 1	0.016 ± 0.001	40 ± 35	0.021 ± 0.007	140 ± 30	0.050 ± 0.004	17 ± 5	0.016 ± 0.001
**2CE**	10 ± 2	0.024 ± 0.001	3.4 ± 0.9	0.0075 ± 0.0004	17 ± 7	0.019 ± 0.003	35 ± 15	0.011 ± 0.001	26 ± 5	0.027 ± 0.002
**2BS**	78 ± 23	0.019 ± 0.002	0.04 ± 0.07	0.021 ± 0.001	1.8 ± 0.3	0.033 ± 0.001	13 ± 3	0.0149 ± 0.0009	6 ± 2	0.019 ± 0.001
**2CEH**	29 ± 6	0.021 ± 0.001	0.3 ± 0.2	0.018 ± 0.001	56 ± 25	0.04 ± 0.01	5 ± 2	0.0127 ± 0.0007	77 ± 22	0.022 ± 0.003
**2ABGS**	22 ± 7	0.013 ± 0.001	1.0 ± 0.2	0.0312 ± 0.0008	7 ± 2	0.017 ± 0.001	17 ± 6	0.019 ± 0.002	17 ± 2	0.035 ± 0.001
**2ABGSFT**	84 ± 24	0.018 ± 0.002	1.3 ± 0.6	0.020 ± 0.001	15 ± 3	0.0199 ± 0.0009	98 ± 41	0.022 ± 0.003	22 ± 5	0.026 ± 0.002
**2ABGSFTCEH**	10 ± 3	0.014 ± 0.001	1.0 ± 0.4	0.016 ± 0.001	5 ± 2	0.025 ± 0.002	weak	weak	weak	weak

[a] 'Weak' indicates that perturbation of the spectrum was detected upon addition of ligand but binding parameters could not be quantified.

[b] Data represent the means +/- SD of n = 3 independent experiments.

Appendix Table S7. Effect of tree topology *vs.* evolutionary rate on the sequence identity of inferred ancestors.

P450 form	Amino acid sequence identity across the entire inferred protein sequence		
	New_without_RF vs. Old_without_RF[a]	New_with RF vs. Old_with RF	Old_without RF vs. Old_with RF[b]
2ABGSFTCEH	0.9494	0.9332	0.9818
2CEH	0.9211	0.915	0.9818
2CE	0.8644	0.8623	0.9919
2C	0.9575	0.9534	0.9899
2E	0.9553	0.9553	0.9959
2ABGSFT	0.9433	0.9372	0.9818
2ABGS	0.9352	0.9291	0.9879
2A	0.9332	0.9332	1
2S	0.8988	0.9008	0.9939
2B	0.9206	0.9165	0.9857
2F	0.8758	0.8859	0.9878
2BS	N/A	N/A	0.9818

Mean	0.9231	0.9202	0.9884
SD	0.0314	0.0283	0.0062
p value, 2-tailed heteroscedastic Student's *t*-test vs comparison between trees (without RF)	N/A	0.817819399	3.50955E-05

[a]New denotes an inference obtained using an alternative tree generated using IQTree. Old denotes the inference obtained using the original tree, from which ancestors were characterized.

[b]Inferences with rate files were obtained by using the rate file determined by IQtree while making the new tree.

[c]All data represent percent amino acid identity across the whole sequence of~ 491-494 residues.

[d] N/A indicates not applicable. The CYP2BS node is not present in the new tree but was present in the reference phylogeny of Kirischian et al., 2011 and the tree used for the inference.

Appendix Table S8. Comparison between the sequences of the resurrected ancestral proteins and the respective consensus sequences for equivalent clades

Clade	Identity [a]	Similarity	Residues differing
2A	439/463 (94.8%)	448/463 (96.8%)	24
2B	373/463 (80.6%)	420/463 (90.7%)	90
2C	383/463 (82.7%)	411/463 (88.8%)	80
2E	389/463 (84.0%)	427/463 (92.2%)	74
2F	420/463 (90.7%)	440/463 (95.0%)	43
2S	371/463 (80.1%)	404/463 (87.3%)	92
2BS	338/463 (73.0%)	392/463 (84.7%)	125
2CE	336/463 (72.6%)	386/463 (83.4%)	127
2ABGS	370/463 (79.9%)	407/463 (87.9%)	93
2CEH	331/463 (71.5%)	379/463 (81.9%)	132
2ABGSFT	352/463 (76.0%)	397/463 (85.7%)	111
2ABGSFTCEH	351/463 (75.8%)	387/463 (83.6%)	112

[a] Sequences were compared from the start of the proline region, i.e. excluding N-terminal modifications introduced to facilitate expression in *E. coli.*

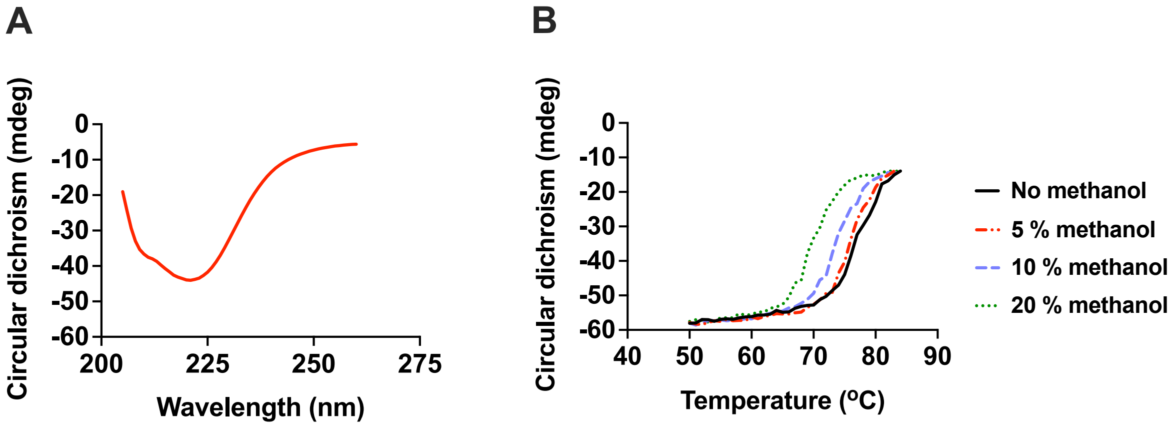

Appendix Figure S1. Analysis of CYP2CEH thermostability by circular dichroism.

Purified CYP2CE (0.4 mg/ml) was analysed in 50 mM sodium phosphate buffer, pH 7.4, using a 1 mm pathlength cuvette on a Jasco J-715 CD spectrometer. A, CD spectrum at 20 °C. B, thermal denaturation curves prepared by monitoring molar ellipticity at 222 nm as the protein was heated in the presence and absence of methanol. The calculated Tm values were: 76.5 °C, no methanol; 75.5 °C, 5 % (v/v) methanol; 73 °C, 10 % (v/v) methanol; and 69 °C, 20% (v/v) methanol.

Thermostability ($^{60}T_{50}$)

P450 Form	A helix buried	A helix exposed	b-sheet 1 buried	b-sheet 1 exposed	B helix buried	B helix exposed	pre B' helix buried	pre B' helix exposed	B' helix buried	B' helix exposed	Post B' helix buried	Post B' helix exposed	C helix buried	C helix exposed
2CEH	8.8	-23.3	24	-0.8	2.9	-2.8	-8.5	-7	11.2	-7.4	11.7	-3.9	2.8	-24.1
2CE	3.7	-23.3	18.9	-4.3	2.9	-2.5	-8.5	-7	8.4	-7.4	16.8	-3.9	5.4	-24.1
2ABGSFTCEH	14.2	-23.3	24	-1.7	2.9	-2.8	-8.5	-7	9.9	-8	11.7	-3.9	2.8	-24.1
2ABGSFT	14.2	-23.3	24	-1.7	2.9	-2.8	-6.6	-7	9.9	-8	11.7	-3.9	2.8	-24.1
2ABGS	14.2	-24	23.6	-1.7	2.9	-2.8	-6.6	-7	8.5	-8	11.4	-3.5	2.8	-24.1
2C	12.7	-20.5	18.9	-4.7	2.9	-2.5	-7.6	-4.3	8.4	-1.7	16.8	-3.9	0.9	-21.5
2BS	16.1	-18.7	23.6	-4.3	2.9	-2.8	-4	-7	11.9	-8	11.4	-3.5	2.8	-24.1
2S	15.1	-14	23.6	-1.7	2.9	-2.8	-4	-7.4	7	-8	1.1	-3.5	0.9	-19.6
2B	11.4	-24.6	21.7	-6.1	2.9	-2.8	-4	-4.2	17.8	-4.3	14.3	-3.5	2.8	-18.8
2C18	4.1	-10.3	17.2	2.8	2.9	-2.5	-7.6	-4.3	5.1	-7.4	9.4	-3.9	6.8	-21.9
2E	8.4	-18	18.5	-5.3	2.9	-3.6	-9.4	-7	1.6	-3.5	-3	-3.9	2.1	-21.5
2C19	7.4	-5.8	13.6	-1.1	5.3	-2.5	-0.6	-6.7	2.3	-8	8.8	-4.5	3.5	-21.9
2F	9.7	-20.8	19	-0.8	2.9	-2.8	-9.8	-7	7.5	-4.8	7.1	-3.9	3.2	-19.2
2A	7.9	-23.1	22.4	-6.1	2.9	-2.8	-12	-7	10.5	-4.4	2	-3.9	6.2	-21.6
2S1	9.5	-13.3	25.6	-2.5	0.1	-4.3	-4.3	-4.2	9.6	-3.9	1.1	-3.5	3.5	-19.6
2B6	10.4	-25.2	17.5	-1.6	8.1	-2.8	0.9	-7.4	13.5	-5.1	14.3	-4.5	1.3	-16.4
2E1	12.6	-19.2	14.7	-4.3	2.9	-3.6	-8.2	-7	1.6	-3.2	-4	-4.5	7.3	-22.2
2F3	13.8	-19.8	20.6	-6.1	2.9	-2.8	-9.8	-7	5.1	-0.7	7.1	-3.9	3.9	-21
2A6	7.9	-23.1	22.4	-6.1	3.4	-2.8	-12	-7	10.9	-4.4	4.4	-3.9	6.2	-21.6
2C8	9	-10.3	16.6	1.9	2.9	-2.5	-14.2	-7	5.9	-8	10.9	-3.9	3.5	-24.5
2C9	7.7	-6.1	16.8	3.8	2.9	-2.5	-0.6	1	2.3	-8	8.8	-4.5	4.1	-21.9
2A13	7.9	-23.1	22.4	-6.1	1	-2.8	-12	-7	10.5	-4.4	2	-3.9	6.2	-18.5
2C11	5.4	-12.6	15.4	1.9	2.9	-2.8	-0.6	-4.3	2.5	-8	9.8	-3.9	5.2	-13.3
2a5	7.9	-23.1	24.6	-6.1	0.7	-2.8	-12	-7	10.5	-4.4	7.1	-3.9	6.2	-21.6

P450 Form	Pre D helix buried	Pre D helix exposed	D helix buried	D helix exposed	Pre E helix buried	Pre E helix exposed	E helix buried	Pre F helix buried	Pre F helix exposed	F helix buried	F helix exposed	F' helix buried	F' helix exposed
2CEH	4.3	-9	1	-22.4	-6.9	-5.5	32.2	-4.3	-10.5	24.5	-16.5	4.2	-10.8
2CE	4.3	-7.8	2	-22	-6.9	-5.5	32.2	-4.3	-10.9	19.8	-16.5	2.5	-8.9
2ABGSFTCEH	4.3	-9	1	-22.4	-6.9	-5.5	32.2	-4.3	-10.5	23.3	-16.5	5.9	-10.8
2ABGSFT	4.3	-9	4.8	-22.4	-1.6	-5.5	25.2	-4.3	-10.5	23.3	-16.5	5.9	-10.8
2ABGS	4.3	-9	4.8	-22.4	-1.6	-5.5	24.2	-4.3	-10.5	21.4	-16.5	4	-9.3
2C	4.3	-9	5.8	-22.8	-7.2	-5.5	31.1	-7.2	-10.9	20.5	-18.6	10.8	-8.6
2BS	4.3	-9	4.8	-22.4	-1.6	-5.5	21.6	-4.3	-10.5	24	-16.1	3	-9.3
2S	5.3	-9	2.5	-19.3	-4.2	-5.5	21.8	-4.3	-10.5	9.8	-10.8	3.4	-9.3
2B	4.3	-9	5.8	-22.4	-0.7	0.3	25.9	-4.3	-10.5	22.3	-11.9	12.1	-3.9
2C18	4.3	-9	5.5	-23.4	-2.3	-5.1	30.8	-9.7	-10.9	12.8	-19.5	13	-8.9
2E	4.3	-7.4	3.4	-23.1	-6.9	-5.1	27.4	-6.9	-10.9	18.5	-13.9	4.7	-8.6
2C19	4.3	-9	5.5	-23.4	-2.3	-5.5	31.1	-10	-10.9	14.9	-18.5	10.6	-8.9
2F	4.3	-9	13	-19.7	-1.6	-5.1	23	-4.3	-7.8	22.9	-15.4	9.5	-8.5
2A	6.4	-9	12.1	-15.8	0.1	-1.7	5.3	-4.3	-10.5	21	-8.3	7.2	-10.8
2S1	5.3	-9	8.3	-13.6	-7.9	-5.1	23.4	-4.3	-0.5	17.7	-6.3	0.4	-11.6
2B6	4.3	-9	5.8	-22.4	-2.6	-0.1	25.9	-4.3	-10.6	24.5	-14.2	10.2	2.8
2E1	2.8	-7.4	3.4	-20.4	-6.9	-5.1	27	-6.9	-10.9	18.8	-16.4	3.9	-8.6
2F3	4.3	-9	10.9	-22.1	-7.3	-5.1	23.7	-4.3	-7.8	23.6	-15.4	6.8	-8.5
2A6	6.4	-9	12.1	-15.8	0.1	-3.9	9.4	-4.3	-10.9	30.8	-8.3	4.6	-10.2
2C8	4.3	-9	5.5	-22.1	-2.3	-5.5	30.5	-7.2	-11.3	11.2	-16.1	11.4	-5.1
2C9	4.3	-9	5.5	-23.4	-2.3	-5.5	31.1	-9.7	-11.3	14.5	-17.9	10.5	-8.9
2A13	6.4	-9	12.1	-15.8	0.1	-6.7	9.4	-4.3	-10.5	25.5	-8.3	7.2	-10.2
2C11	4.3	-9	5.9	-22.4	-1.7	-5.5	31.1	-10	-10.9	9.5	-13.5	14.8	-8.9
2a5	6.4	-9	8.5	-19.3	0.1	-0.7	5.3	-4.3	-10.5	25.5	-8.3	7.2	-7.6

P450 Form	G helix buried	G helix exposed	Pre H helix buried	Pre H helix exposed	H helix buried	H helix exposed	Pre I helix buried	Pre I helix exposed	I helix buried	I helix exposed	J helix buried	J helix exposed
2CEH	8.9	-34.7	-1.3	-14.6	15.4	-7	-1.5	-22.7	20.7	-1.6	8.8	-22.4
2CE	10.9	-39.4	-1.3	-14.6	15.4	-7	-1.5	-23.4	18.1	-1.6	8.8	-22.4
2ABGSFTCEH	8.9	-34.4	0.5	-14.6	15.4	-7	-1.5	-22.7	20.7	-1.6	8.8	-22.4
2ABGSFT	8.9	-34.4	0.5	-14.6	15.4	-7	-1.5	-22.7	20.7	-1.6	8.5	-22.4
2ABGS	9.9	-34	0.5	-14.6	15.4	-7	-1.5	-22.7	24.9	-2	8.5	-22.4
2C	10.8	-39.4	-1.3	-17.3	15.4	-7	-1.5	-19.9	20.7	-1.6	9.4	-22.4
2BS	9.9	-34.3	0.5	-11.9	14.7	-7.4	-1.5	-22.7	28.8	-7.1	8.5	-22.4
2S	16.9	-30.7	0.5	-11.9	13.7	-7.4	3.5	-22.7	36.1	-7.1	-0.6	-22.4
2B	13.4	-37.8	-1.3	-11.9	10.8	-7.4	-1.5	-20	29.5	-7.1	13.5	-22.4
2C18	6.5	-29.1	2.1	-11.2	15.4	-7	-1.5	-21.8	19.2	0.7	10.7	-10.1
2E	5.8	-38	-1.3	-17.3	11.6	-7.4	0.8	-22.7	25.2	0.3	6.2	-22.4
2C19	5	-25.4	-3.5	-11.3	15.4	-7.4	-1.5	-22.1	24.1	1	10.6	-17.1
2F	11	-34.1	-1.3	-14.7	15.4	-1.7	-1.2	-15.4	18.7	-1.6	7.9	-18.1
2A	7.6	-37.2	-1.3	-14.6	11.5	-7	-1.4	-21.2	23	-2	6.6	-17.1
2S1	14.8	-25.7	0.5	-10.9	14.6	-1.7	-1.4	-16.7	40.1	-7.4	-1.9	-19.5
2B6	4	-35.7	-1.3	-8.7	9.1	-7.4	-1.5	-17.5	12.6	-6.7	15.7	-21.7
2E1	3.6	-35.1	-1.3	-11.4	12.3	-7.4	-9.6	-17.4	23.9	-1.6	8.4	-22.4
2F3	6.9	-35.5	-1.3	-14.7	10.2	-1.7	-1.2	-9.4	10.3	-1.6	7.9	-15.3
2A6	13.9	-36.5	-1.3	-14.6	11.5	-7	-1.4	-20.8	22.5	-0.1	6.6	-17.1
2C8	-3.9	-19.1	-1.3	-11.6	15.4	-7	-1.5	-25.3	23	1	9.1	-15.8
2C9	6.9	-25.4	-3.5	-12.9	12.8	-7.4	-1.5	-19.9	12.5	1	9.1	-17.1
2A13	6.6	-36.9	-1.3	-14.6	11.5	-7	-1.4	-20.8	23	-0.1	6.6	-17.1
2C11	8.5	-30.8	-1.3	-19.7	3.6	-7	-1.5	-19.9	18.9	0.3	14.9	-17.1
2a5	5.1	-36.9	-1.3	-14.6	11.5	0.3	-1.4	-21.2	24.9	-2	6.9	-17.1

P450 Form	J' helix buried	J' helix exposed	K helix buried	b-sheet 2 buried	b-sheet 2 exposed	K' helix and buried unstructured regio buried	K' helix and buried unstructured regio exposed	L helix buried	L helix exposed	b-sheet 3 buried	b-sheet 3 exposed
2CEH	-7	-21.5	0.5	20	-21.8	16.1	-43.3	19.5	-3.9	16.1	-22.5
2CE	-3.7	-15.8	0.5	19.3	-18.7	19.1	-43.2	19.5	-3.9	15.4	-22.9
2ABGSFTCEH	-7	-21.5	0.5	17.8	-21.8	16.1	-38	18.5	-3.9	17.1	-22.5
2ABGSFT	-7	-22.1	0.5	15.3	-21.8	16.1	-38	18.5	-3.9	16.8	-22.5
2ABGS	-7	-22.1	-4.8	15.7	-21.8	15.7	-41.7	18.5	-3.9	23	-25.6
2C	-3.7	-18.8	-3.9	20.6	-20.1	18.1	-42.9	19.5	-3.9	7.6	-14.1
2BS	-7	-16.9	-4.8	14.3	-21.2	15.7	-36.4	15.9	-3.9	22.3	-22.1
2S	-3.2	-10.7	-1.5	18.3	-19.3	22.1	-41.3	15.9	-3.9	10	-12.9
2B	-2.5	-16.3	-4.8	10.9	-23.7	21	-32.7	15.9	-3.9	21.8	-29.2
2C18	-3.7	-20.8	-3.9	21.5	-13.5	6.7	-37.9	19.5	-3.9	15.9	-10.9
2E	-7	-8.7	2.8	11.3	-16.3	8.1	-40.3	19.5	-3.9	22.4	-17
2C19	-3.7	-20.4	-4.2	21.5	-13.5	11.6	-41.2	23	-3.9	16.1	-4.6
2F	-5	-14.2	-2.5	11.9	-12.3	7.3	-34.3	17.9	-4.3	9.5	-14.1
2A	-9.2	-21.5	-4.4	11.5	-20.5	18.4	-46	19.5	-3.9	25.3	-38.4
2S1	-7	-5.9	-3.9	18	-17.3	27.4	-44.7	19	-3.9	18.7	-18.9
2B6	-7.8	-16.2	-2.9	20.3	-16	19.8	-27.2	19.1	-3.9	7.1	-13.1
2E1	-1.8	-13.3	2.8	12.7	-9.6	-0.6	-46.5	26.2	-3.9	20	-20.7
2F3	-4.6	-13.9	-2.5	15.3	-22.7	12.6	-29.7	17.9	-1.7	6.5	-10.8
2A6	-9.2	-21	-1.8	13.8	-24.7	18.3	-43.3	16.3	-3.9	16.4	-39
2C8	-3.7	-20.5	-9.2	19.4	-17.3	13.8	-38	19.5	-3.9	24	-15.8
2C9	-3.7	-20.8	-8.7	26.3	-14.1	11.6	-40.3	23.5	-1.7	16.1	-4.9
2A13	-9.2	-21	-4.4	12.1	-23.9	20.5	-48.4	16.6	-3.9	15.4	-39.5
2C11	-3.7	-21.5	-3.9	23.3	-22.1	9.4	-47	15.9	-1.7	18.6	-7.3
2a5	-13.3	-21.5	-2.2	11.9	-20.5	18.4	-47.2	14.8	-3.9	19.7	-31.3

100%

50%

0%

Appendix Figure S2. Kyte-Doolittle hydrophobicity of buried and exposed residues in specific structural elements.

The figure shows hydrophobicity values for buried and exposed residues in each of 26 structural regions listed from the N- to the C-terminal. Colours represent relative hydrophobicity, from most hydrophobic (red) to most hydrophilic (blue). The heatmap refers to the scale within each column and is scaled from 0-100% of the range of hydrophobicity.

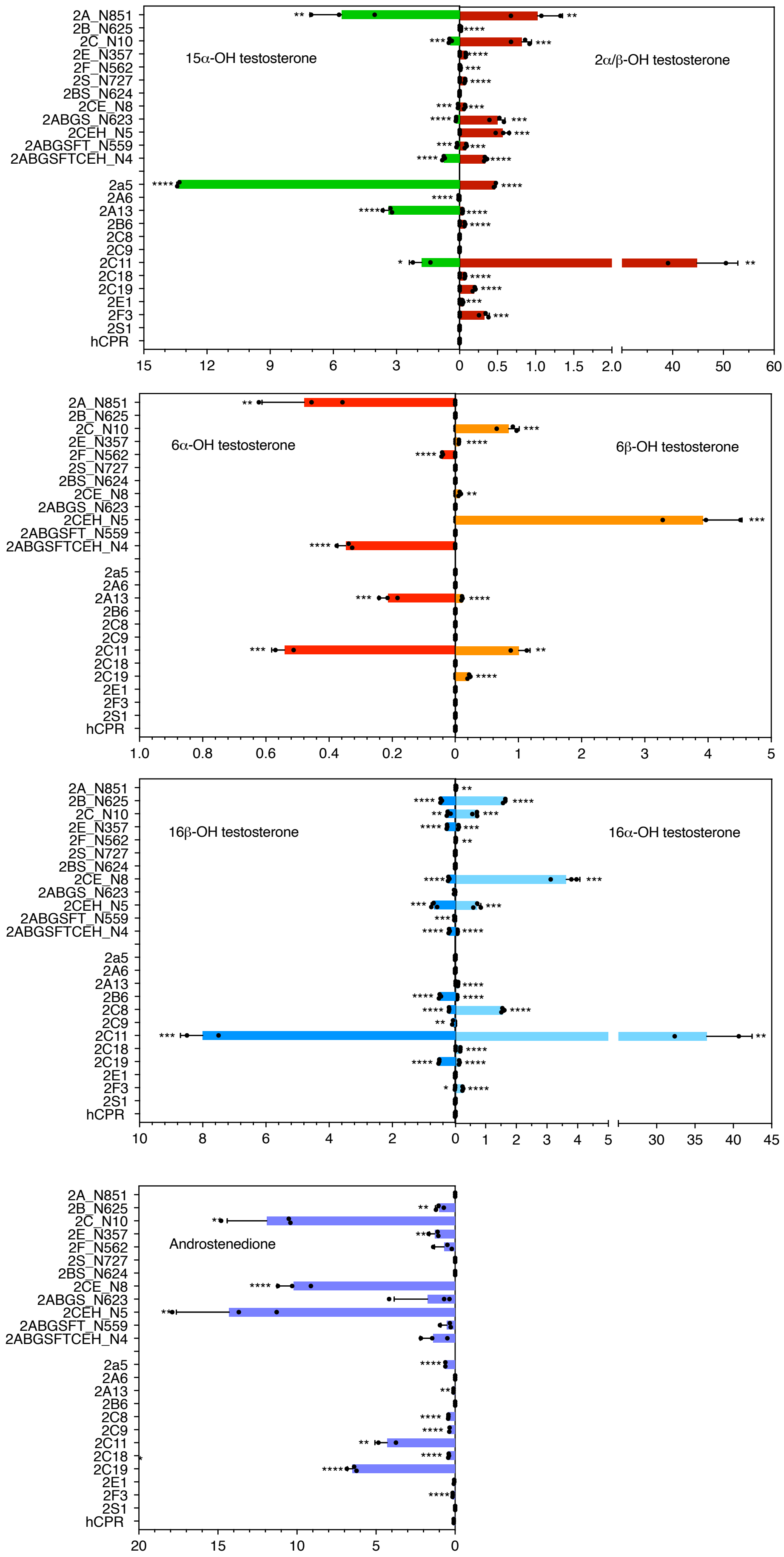
15α-OH testosterone
2α/β-OH testosterone
6α-OH testosterone
6β-OH testosterone
16β-OH testosterone
16α-OH testosterone
Androstenedione
2A_N851
2B_N625
2C_N10
2E_N357
2F_N562
2S_N727
2BS_N624
2CE_N8
2ABGS_N623
2CEH_N5
2ABGSFT_N559
2ABGSFTCEH_N4
2a5
2A6
2A13
2B6
2C8
2C9
2C11
2C18
2C19
2E1
2F3
2S1
hCPR

Appendix Figure S3. Metabolism of testosterone by the CYP2 extant and ancestral enzymes.

Total production of six hydroxylated metabolites (6α-, 6β-, 15α, 16α, 1cβ and 2α/β-hydroxytestosterone) as well as androstenedione. Reactions were carried out using 100 μM testosterone and 0.5 μM P450 in bacterial membranes containing co-expressed hCPR. Reactions were initiated with the addition of an NADPH-generating system and incubated for 2 hr at 37 °C. Products were separated by HPLC and where possible, metabolites were identified and quantified by comparison to authentic standards. In the case of 15α-hydroxytestosterone, 2A_N851 was used to generate a sufficient quantity for identification by NMR. 15α-hydroxytestosterone was quantified using the standard curve for 15β-hydroxytestosterone, assuming they showed approximately equivalent absorbance at the wavelength chosen. Data represent the mean ± SD of n=3 experiments. Asterisks indicate a significant increase in the respective metabolite formation over the negative control, i.e., membranes from *E. coli* expressing hCPR but no P450; * $p<0.05$, ** $p<0.01$, *** $p<0.001$, **** $p<0.0001$, two-tailed Student's *t*-test.

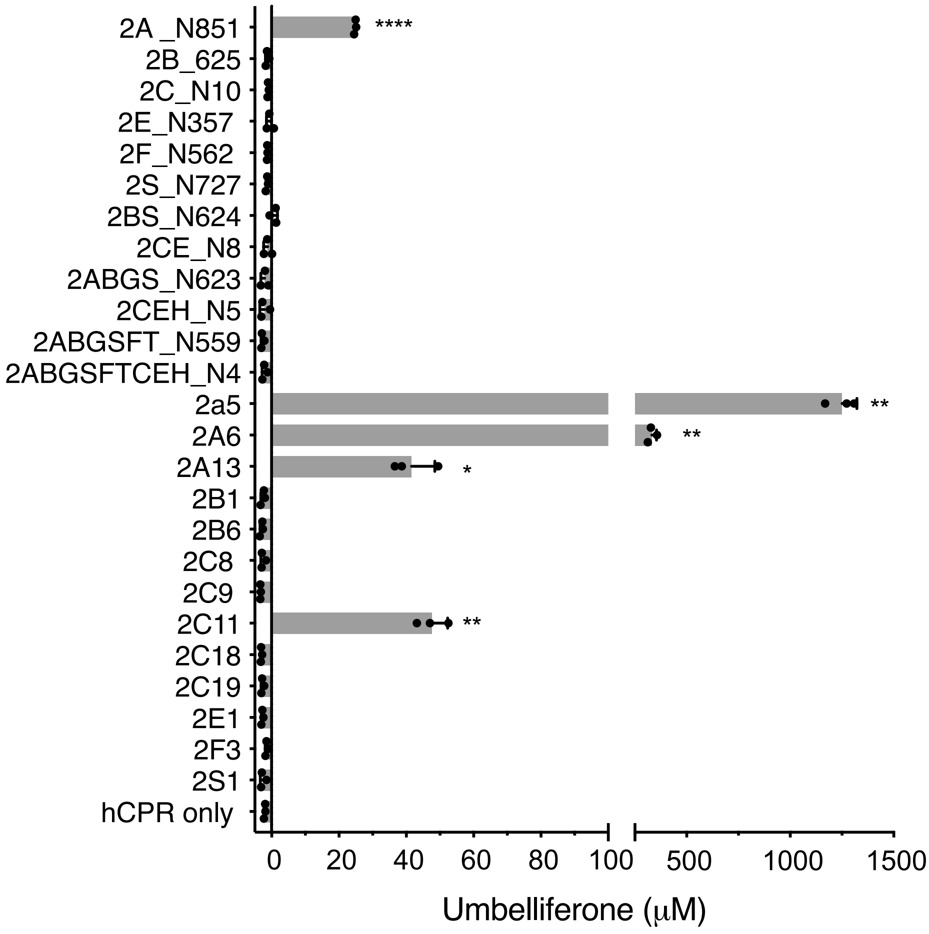

Appendix Figure S4. Conversion of coumarin to umbelliferone (7-hydroxycoumarin) by the CYP2 extant and ancestral enzymes.

Reactions were performed using bacterial membranes containing co-expressed P450 and hCPR. Incubations contained 0.1 μM P450, 50 μM coumarin and an NADPH-generating system (NGS) in a reaction volume of 250 μl. Reactions were carried out at 37 ºC for 10 min and the products extracted as described previously [46]. The 7-hydroxylated product (umbelliferone) was detected via its fluorescence using excitation/emission wavelengths of 358 and 454 nm, respectively. Fluorescence was converted to umbelliferone concentration using an umbelliferone standard curve. Data represents the mean ± SD of n = 3 reactions for each form. Asterisks indicate significantly higher production of umbelliferone than in the hCPR control: *, p<0.01; **, p<0.005; ***, p<0.001; ****, p<0.0001; two-tailed Student's *t*-test.

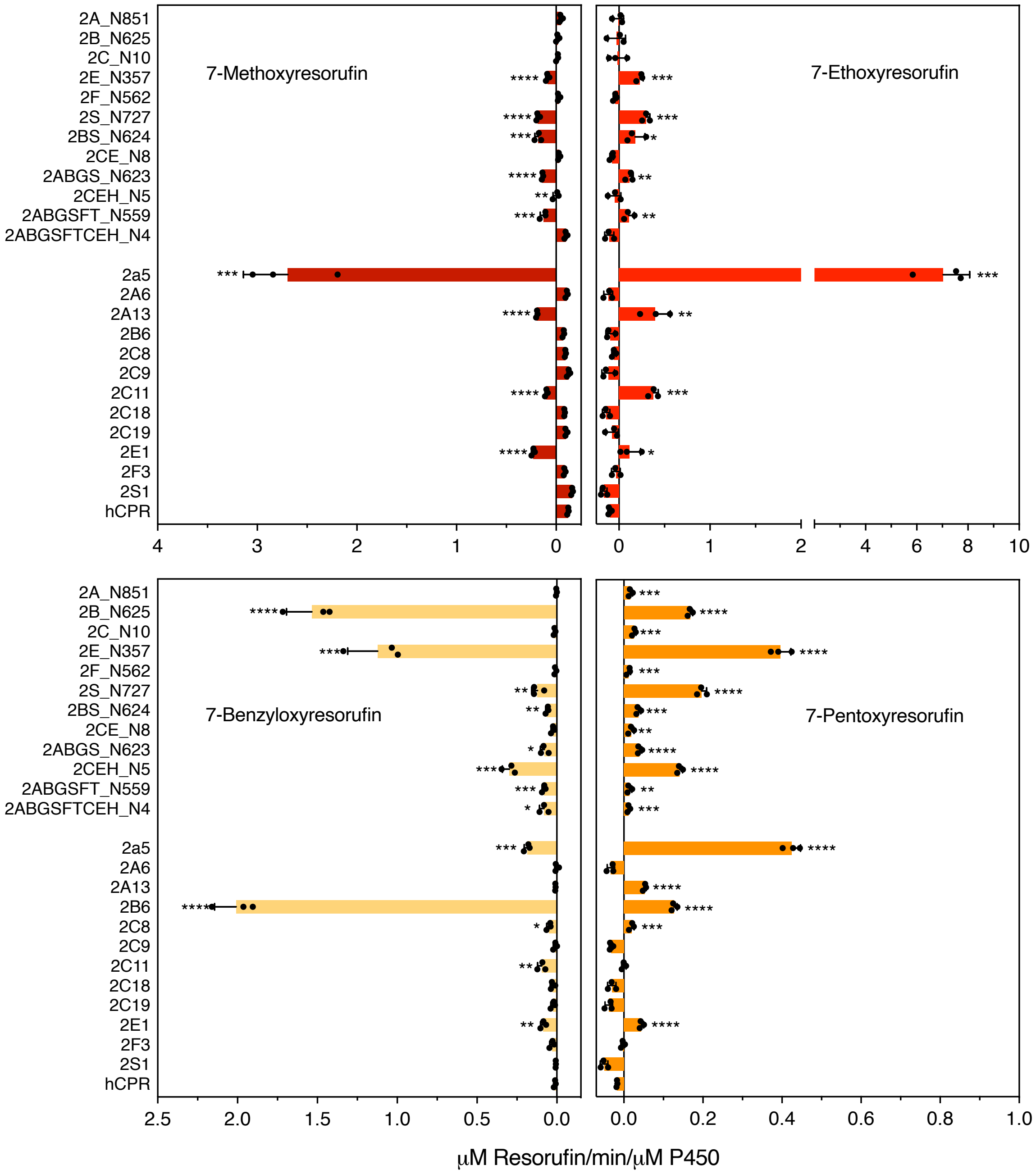

Appendix Figure S5. ***O*-Dealkylation of fluorogenic 7-alkoxyresorufin derivatives by CYP2 extant and ancestral enzymes.**

The *O*-dealkylation of 7-methoxy-, 7-ethoxy-, 7-pentoxy-, and 7-benzyloxy resorufin by ancestral and extant P450s was assessed. Reactions were carried out using 5 nM P450 in bacterial membranes containing co-expressed hCPR. At this concentration, the initial rate

was determined to be in the linear range for the most active forms for each derivative. Membranes were incubated with 5 µM substrate at 37 °C for 3 min at which point the reaction was initiated by the addition of an NADPH-generating system. Data represents the mean ± SD of n=3 reactions. Asterisks indicate a significant increase in the rate of activity over the negative control, i.e., membranes from *E. coli* expressing hCPR but no P450; * $p<0.05$, ** $p<0.01$, ** $p<0.001$, **** $p<0.0001$, two-tailed Student's *t*-test.

A

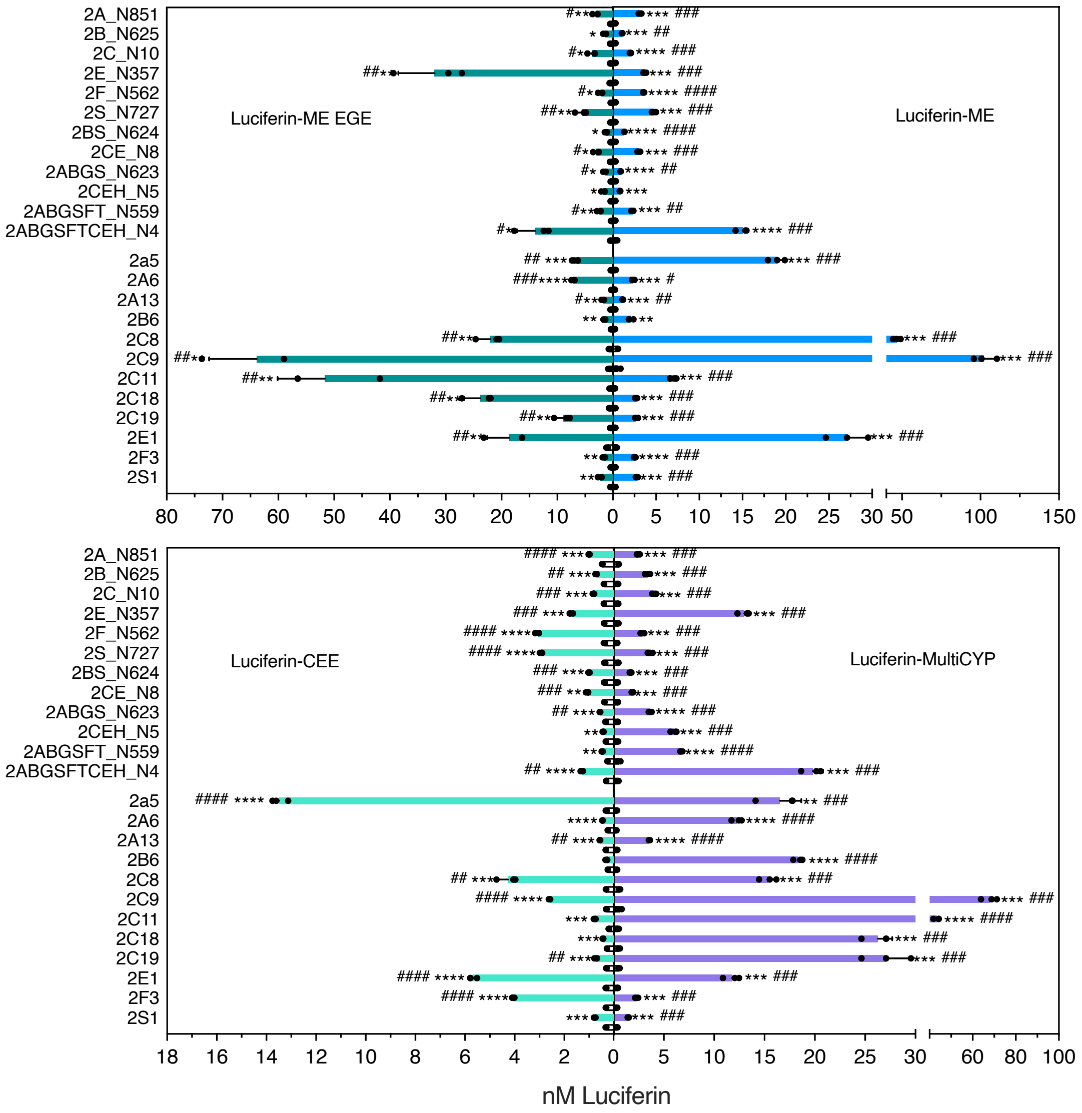

2A_N851
2B_N625
2C_N10
2E_N357
2F_N562
2S_N727
2BS_N624
2CE_N8
2ABGS_N623
2CEH_N5
2ABGSFT_N559
2ABGSFTCEH_N4
2a5
2A6
2A13
2B6
2C8
2C9
2C11
2C18
2C19
2E1
2F3
2S1
Luciferin-ME EGE
Luciferin-ME
Luciferin-CEE
Luciferin-MultiCYP
80 70 60 50 40 30 20 10 0 5 10 15 20 25 30 50 100 150
18 16 14 12 10 8 6 4 2 0 5 10 15 20 25 30 60 80 100
nM Luciferin

B

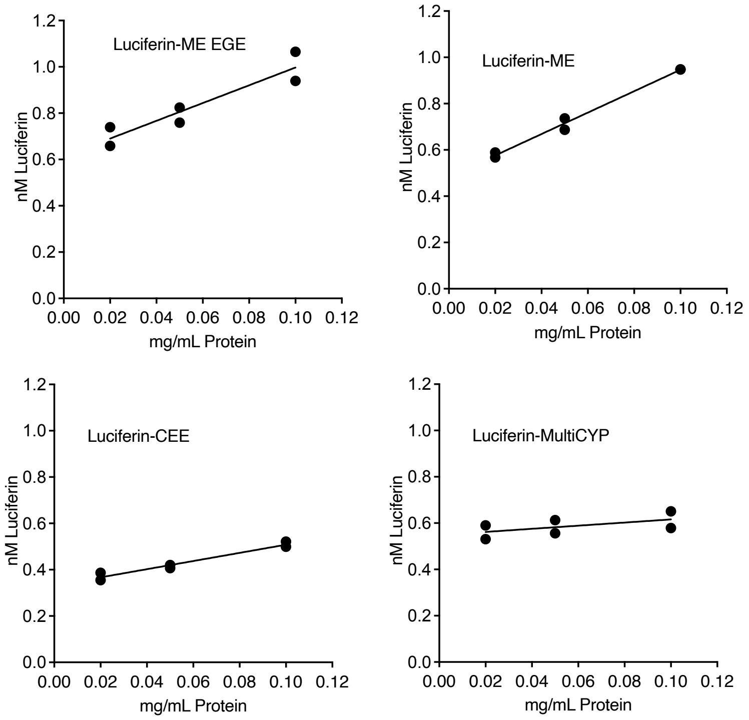

Luciferin-ME EGE
Luciferin-ME
Luciferin-CEE
Luciferin-MultiCYP
nM Luciferin
mg/mL Protein
0.0 0.2 0.4 0.6 0.8 1.0 1.2
0.00 0.02 0.04 0.06 0.08 0.10 0.12

Appendix Figure S6. Metabolism of P450-Glo^{TM} luciferin derivatives by ancestral and extant CYP2 enzymes.

A, Formation of luciferin from luciferin-ME EGE, luciferin-ME, luciferin-CEE and luciferin-MultiCYP. Reactions were carried out using 40nM P450 in bacterial membranes containing co-expressed hCPR. Substrates were used at concentrations of 30 µM (luciferin-ME EGE and luciferin-CEE), 100 µM (luciferin-ME) and 50 µM (luciferin-CEE). Reactions were initiated by the addition of an NADPH-generating system and incubated at 37 °C for 60 min before the addition of the luciferin detection reagent. The luciferin product was detected by luminescence and quantified using a standard curve prepared with luciferin. Data represent the mean ± SD of n=3 reactions with (coloured bars) and n=2 without the NADPH-generating system (open bars). Asterisks indicate significantly higher substrate turnover in the presence of NADPH compared to samples lacking the cofactor; * $p<0.05$, ** $p<0.01$, *** $p<0.001$,**** $p<0.0001$, two-tailed Student's t-test. Hash symbols indicate where turnover by a given form is significantly higher than activity seen using membranes containing hCPR and noP450 at an equivalent total protein concentration; # $p<0.05$, ## $p<0.01$, ### $p<0.001$, #### $p<0.0001$, two-tailed Student's t-test. B, the predicted background luciferin production (nM)at the protein concentration for each form was interpolated from the appropriate standard curve relating apparent activity seen in the P450-deficient ('hCPR') negative control.

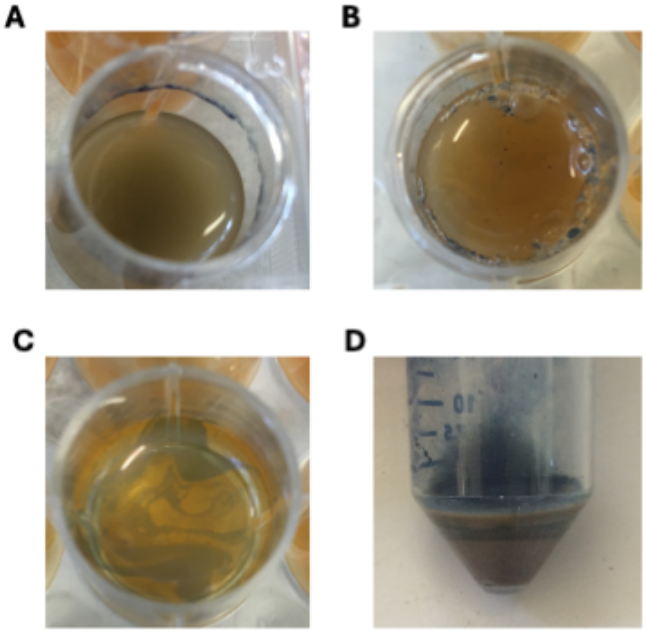

Appendix Figure S7. Indigo formation by ancestral CYP2s. *E. coli* cultures co-expressing ancestral P450s, CYP2A_N851 (A), CYP2E_N357 (B) CYP2CEH_N5 (C) and CYP2F_N562 (D) with hCPR that showed signs of indigo formation, indicating hydroxylation of indole.

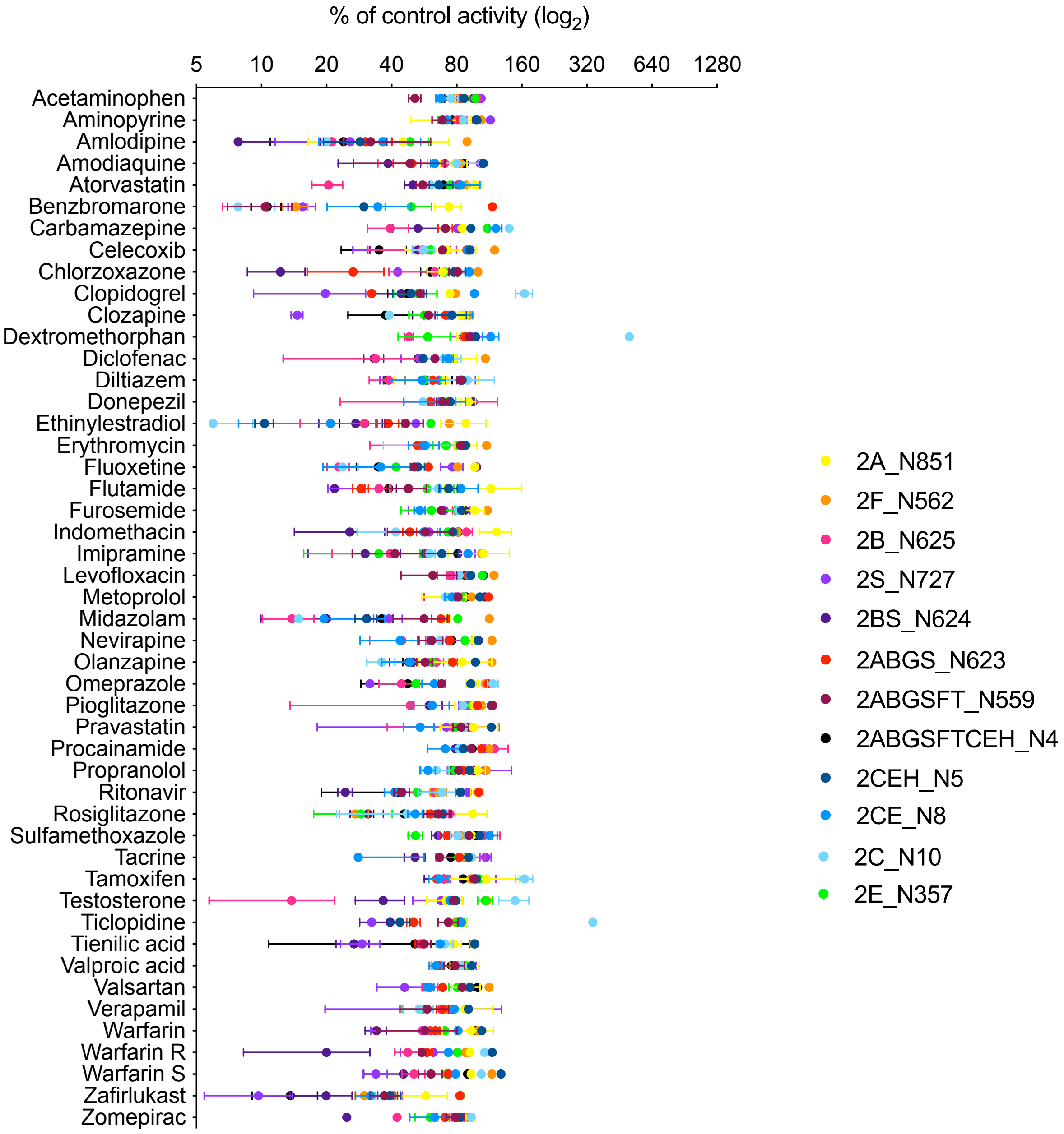

Appendix Figure S8. Effect of alternative ligands on the activity of ancestral CYP2 forms.

The plot shows the relative percentage activity of each ancestor towards the respective probe substrate in the presence of 50 µM of each of the compounds indicated on the y-axis. Full screens were carried out as single measurements with a second measurement carried out on compounds affecting activity at levels greater than 20%. Where shown, error bars represent the range of the two independent replicate measurements.

Reference

Sennett MA, Theobald DL. 2023. Extant Sequence Reconstruction: The accuracy of ancestral sequence reconstructions evaluated by extant sequence cross-validation. bioRXiv:2022.2001.2014.476414.
