# Supplementary material for: Thermostable ancestors enabled evolutionary diversification of promiscuous chemical defence enzymes
Source: EMBO J. 2026 Mar 26;45(9):2938–64. doi: 10.1038/s44318-026-00699-y (PMC13144609; doi:10.1038/s44318-026-00699-y)
Supplement: Supplementary file 8 — Expanded View Figures [file 44318_2026_699_MOESM8_ESM.pdf]

Expanded View Figures

Figure EV1. CYP2 phylogenetic tree.

The CYP2 clade used for reconstruction incorporates the tetrapod subfamilies, CYP2A, CYP2B, CYP2C, CYP2E, CYP2F, CYP2G, CYP2H, CYP2S and CYP2T, and was inferred from 975 extant CYP2 sequences. Sequences were collected from NCBI and Uniprot databases using a BLAST-search of sequences with >40% similarity to characterized CYP2 forms. Phylogenetic relationships were reconstructed via the T-REX web server (Boc et al, 2012), using the maximum likelihood (ML) method under the Jones–Taylor–Thornton (JTT) substitution model (Jones et al, 1992) with PhyML (Guindon and Gascuel, 2003). The final tree was inferred using PhyML. Bootstrapping analysis with 100 replicates was performed to evaluate the tree. The CYP2M subfamily was used as an outgroup to root the tree. Nodes are labeled with bootstrap values. Extant forms are labeled with a five letter code comprising the first three letters of the genus and two letters of the species name, e.g., HOMSA for *Homo sapiens*.

 © The Author(s)

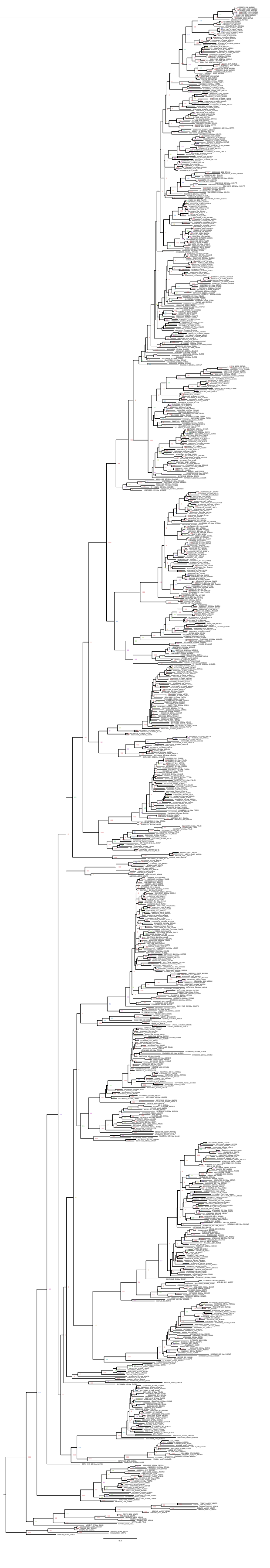

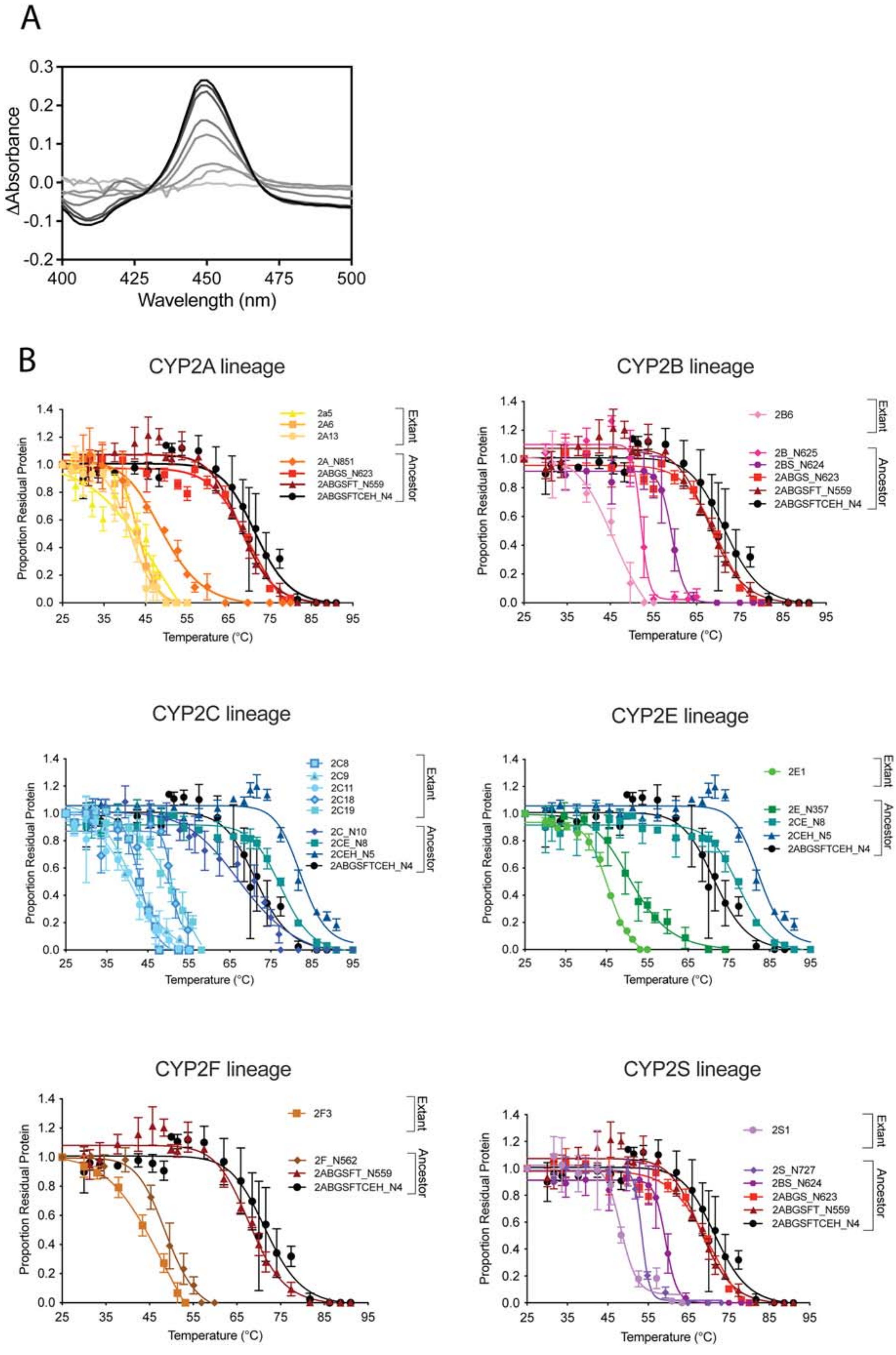
A
ΔAbsorbance
Wavelength (nm)
B
CYP2A lineage
2a5
2A6
2A13
2A_N851
2ABGS_N623
2ABGSFT_N559
2ABGSFTCEH_N4
Extant
Ancestor
CYP2B lineage
2B6
2B_N625
2BS_N624
2ABGS_N623
2ABGSFT_N559
2ABGSFTCEH_N4
CYP2C lineage
2C8
2C9
2C11
2C18
2C19
2C_N10
2CE_N8
2CEH_N5
2ABGSFTCEH_N4
CYP2E lineage
2E1
2E_N357
2CE_N8
2CEH_N5
2ABGSFTCEH_N4
CYP2F lineage
2F3
2F_N562
2ABGSFT_N559
2ABGSFTCEH_N4
CYP2S lineage
2S1
2S_N727
2BS_N624
2ABGS_N623
2ABGSFT_N559
2ABGSFTCEH_N4
Proportion Residual Protein
Temperature (°C)

© The Author(s)

Figure EV2. Thermal stability profiles of extant and ancestral variants of CYP2 enzymes.

Bacterial cells expressing each enzyme were heated for 60 min at temperatures between 25 °C and 100 °C, followed by 5 min at 4 °C and equilibration to room temperature for 5 min. The P450 content was then measured by Fe(II).CO *vs*. Fe(II) difference spectroscopy. (**A**) Representative spectra show the loss of the peak at 450 nm in samples heated at increasing temperatures from 25 °C (black) to 65 °C (light gray). (**B**) The P450 remaining intact in heated samples was calculated as a proportion of total P450 in a sample left at 25 °C. Data represent the mean ± SD of $n = 3$ biological replicates. Each set of plots compares the forms that are related along a single lineage as indicated. All ancestors showed significantly different $^{60}T_{50}$values to their immediate ancestor along the same lineage except CYP2ABGSFT (compared to CYP2ABGSFTCEH) and CYP2ABGS (compared to CYP2ABGSFT); two-tailed, heteroscedastic Student's *t* test. All extant enzymes showed $^{60}T_{50}$ values that were significantly lower than all their cognate ancestors ($P < 0.006$, two-tailed heteroscedastic Student's *t* test). A detailed statistical analysis of the significance of differences in $^{60}T_{50}$ values can be found in Appendix Table S3.

© The Author(s)

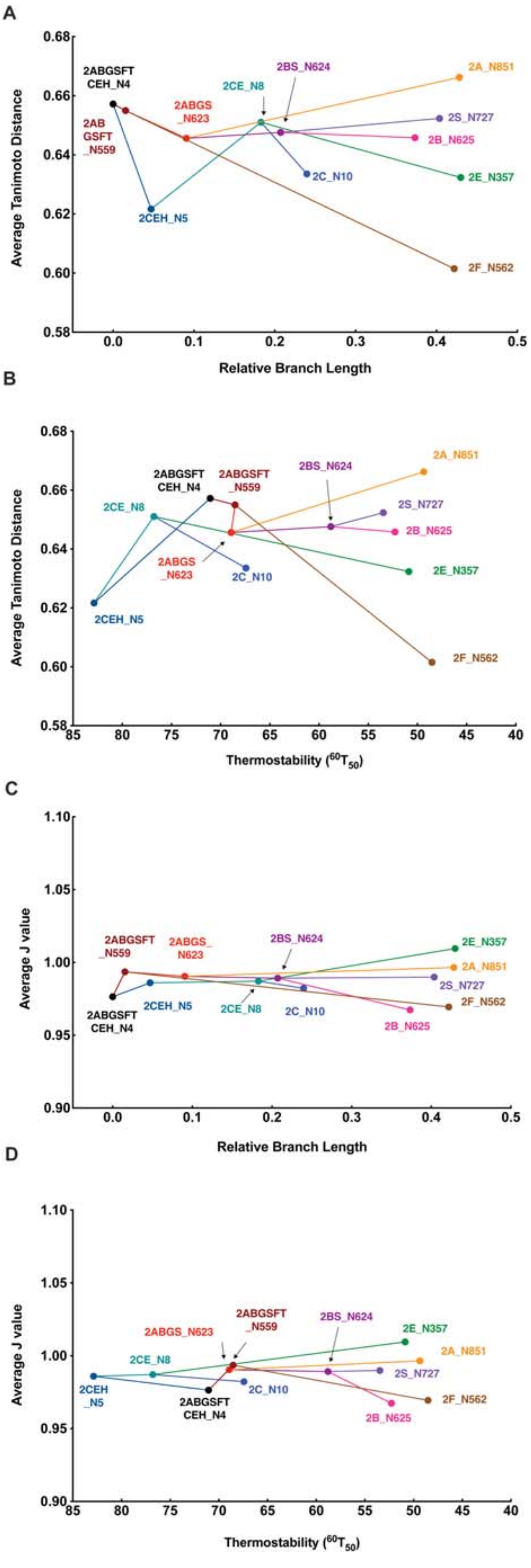
A
0.68
0.66
0.64
0.62
0.60
0.58
Average Tanimoto Distance
0.0
0.1
0.2
0.3
0.4
0.5
Relative Branch Length
2ABGSFT CEH_N4
2AB GSFT _N559
2ABGS _N623
2CEH_N5
2CE_N8
2BS_N624
2C_N10
2A_N851
2S_N727
2B_N625
2E_N357
2F_N562
B
0.68
0.66
0.64
0.62
0.60
0.58
Average Tanimoto Distance
85
80
75
70
65
60
55
50
45
40
Thermostability ($^{60}T_{50}$)
2ABGSFT CEH_N4
2ABGSFT _N559
2CE_N8
2ABGS _N623
2CEH_N5
2C_N10
2BS_N624
2A_N851
2S_N727
2B_N625
2E_N357
2F_N562
C
1.10
1.05
1.00
0.95
0.90
Average J value
0.0
0.1
0.2
0.3
0.4
0.5
Relative Branch Length
2ABGSFT _N559
2ABGS_ N623
2ABGSFT CEH_N4
2CEH_N5
2CE_N8
2C_N10
2BS_N624
2E_N357
2A_N851
2S_N727
2F_N562
2B_N625
D
1.10
1.05
1.00
0.95
0.90
Average J value
85
80
75
70
65
60
55
50
45
40
Thermostability ($^{60}T_{50}$)
2ABGS_N623
2ABGSFT _N559
2CE_N8
2CEH _N5
2ABGSFT CEH_N4
2C_N10
2BS_N624
2E_N357
2A_N851
2S_N727
2F_N562
2B_N625

© The Author(s)

Figure EV3. Ligand promiscuity of CYP2 ancestors measured by average Tanimoto distances and *J* values.

The ligand set of any given enzyme was defined by compounds that activated or inhibited the P450's activity by >20% (Appendix Fig. S8 and Appendix Table S5). The similarity between all compounds in the ligand set was quantified using the Tanimoto distance measure, i.e., for a pair of compounds A and B, where *a* is the number of features present only in A, *b* is the number of features present only in B, and *c* is the number of features present in both A and B, the Tanimoto distance is defined as $T_{ab} = \frac{c}{a+b-c}$. The chemical features of each compound were defined by the MACCS keyset of 166 chemical descriptors. The distance measure between any two compounds was defined as $1 - T_{ab}$ such that greater dissimilarity between two compounds equated to a value closer to 1. The ligand promiscuity of each enzyme was quantified as described in Nath and Atkins (2008) and Foti et al (2011) except that the effect of an inhibitor or activator on a given form was quantified as the proportional change in activity in the presence of the effector, independent of the direction of the effect. (**A**) shows the relationship between average Tanimoto distance and the evolutionary distance between forms (relative branch length). (**B**) shows the relationship between average Tanimoto distance and thermostability ($^{60}T_{50}$). (**C**) shows the relationship between average J value and the evolutionary distance between forms (relative branch length). (**D**) shows the relationship between average J value and thermostability ($^{60}T_{50}$).

© The Author(s)

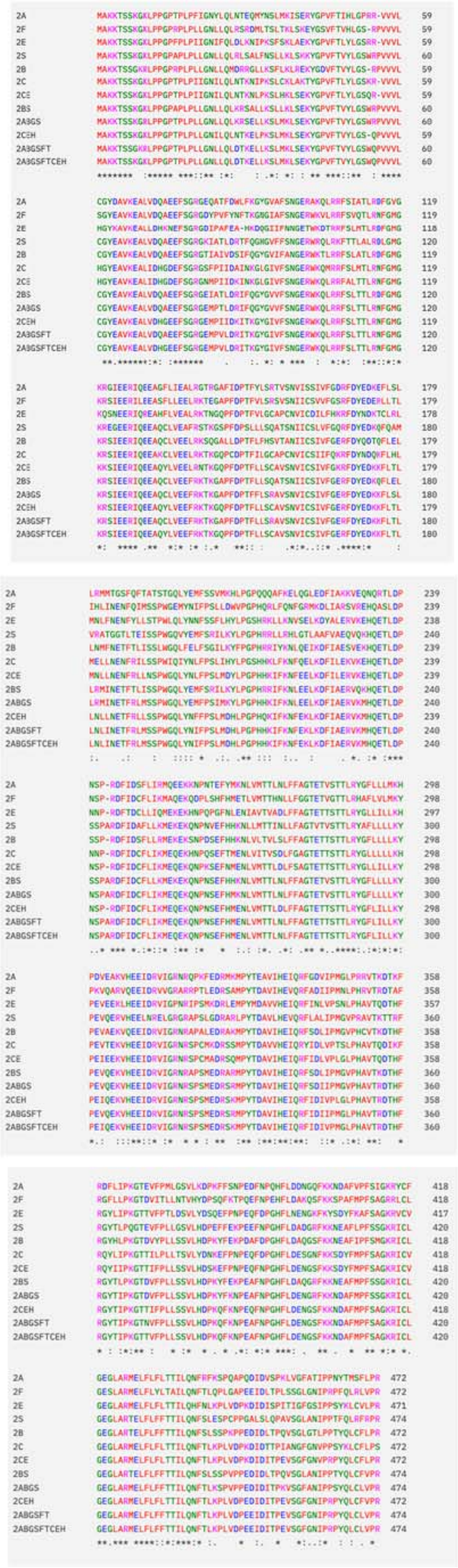

 © The Author(s)

Figure EV4. Alignment of the ancestral CYP2 sequences resurrected herein.

Sequences are shown in the form in which they were characterized, including the modifications made to the N-terminal membrane anchor to enable expression in *E. coli*.
